# Supplementary figures and images for: Evolutionary dynamics in the Anthropocene: Life history and intensity of human contact shape antipredator responses
Source: PLoS Biol. 2020 Sep 22;18(9):e3000818. doi: 10.1371/journal.pbio.3000818 (PMC7508406; doi:10.1371/journal.pbio.3000818)

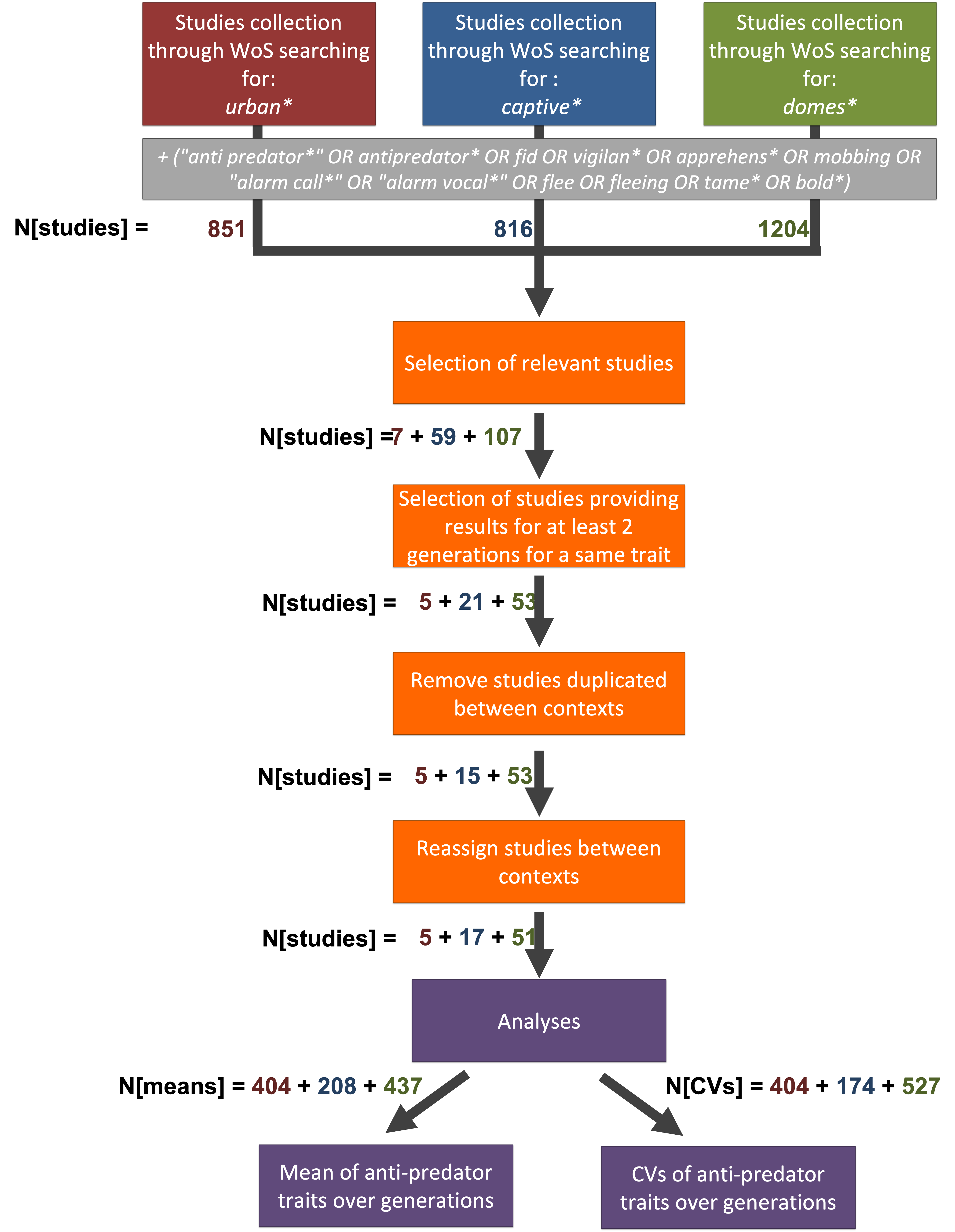

Supplement: S1 PRISMA Checklist — The orange rectangles narrow the numbers of studies per context prior to the analysis steps (purple rectangles) performed on the multiple means and CVs of antipredator traits within each study. Numbers are provided per context: red for urbanization; blue for captivity; and green for domestication. CV, coefficient of variation; PRISMA, Preferred Reporting Items for Systematic Reviews and Meta-Analyses (TIF) [file pbio.3000818.s001.tif]

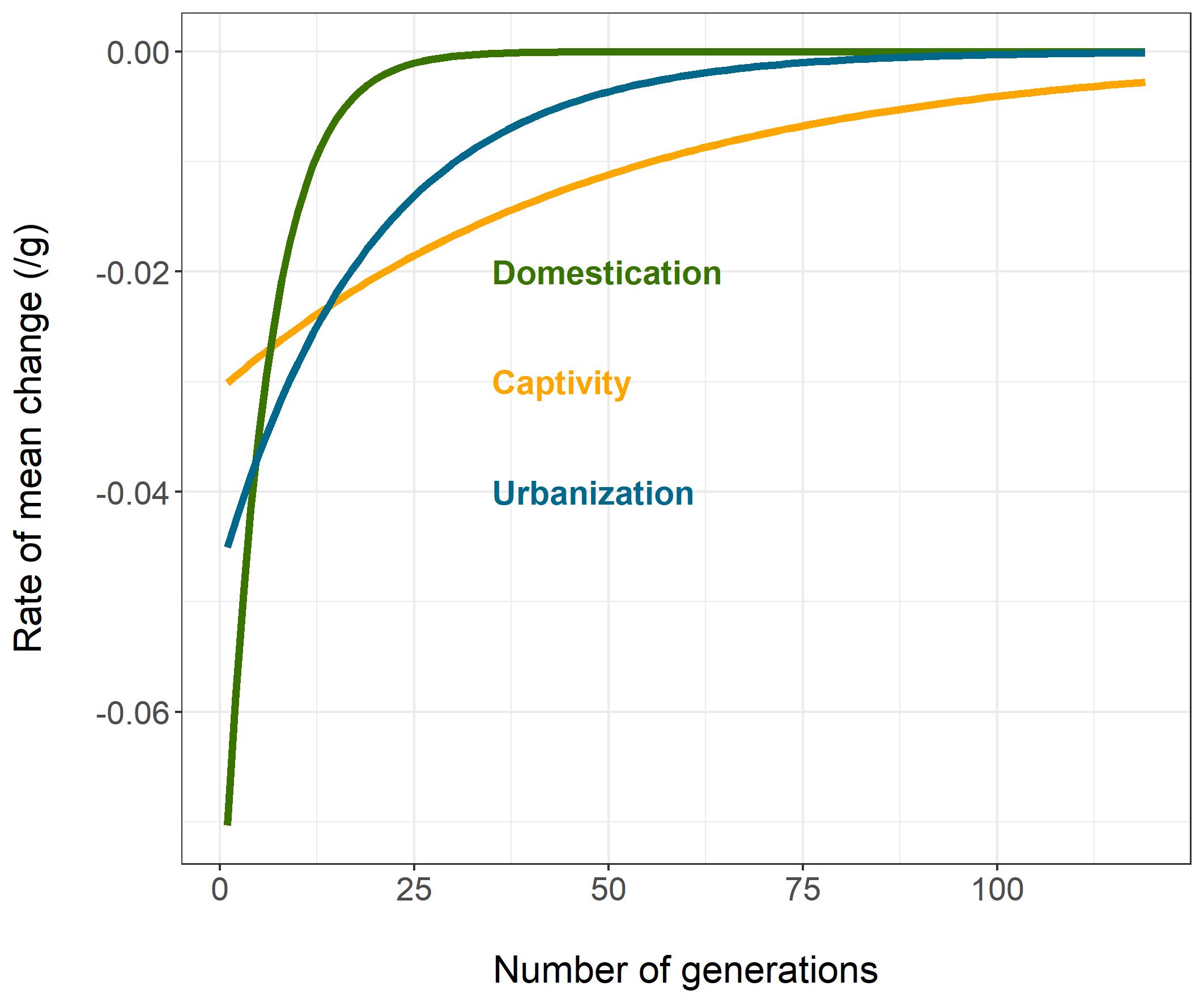

Supplement: S1 Fig — Values are expressed as a rate of change per generation (/g). Lines correspond to the derivatives for the best inverse model fitting the data using the cma-es function. All data and R code supporting the figure are available in S1C Data. cma-es, covariance matrix adapting evolutionary strategy. (TIF) [file pbio.3000818.s002.tif]

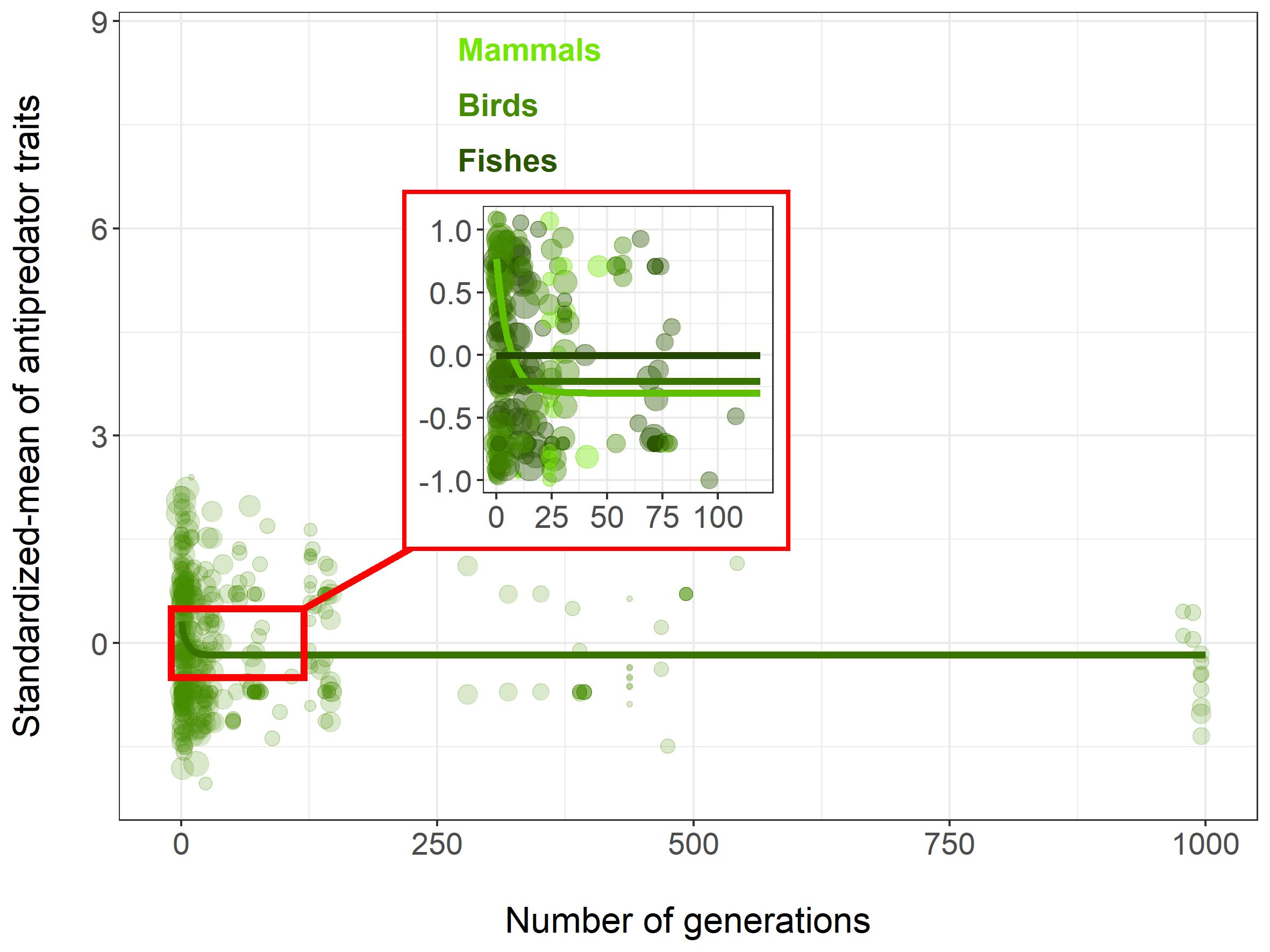

Supplement: S2 Fig — The lines represent the best inverse model with the outputs (slopes and intercepts) of the MCMCglmm. Dot size is proportional to the log-transformed number of replicates used in each study. All data and R code supporting the figure are available in S1C Data. MCMCglmm, Markov chain Monte Carlo generalized linear mixed model. (TIF) [file pbio.3000818.s003.tif]

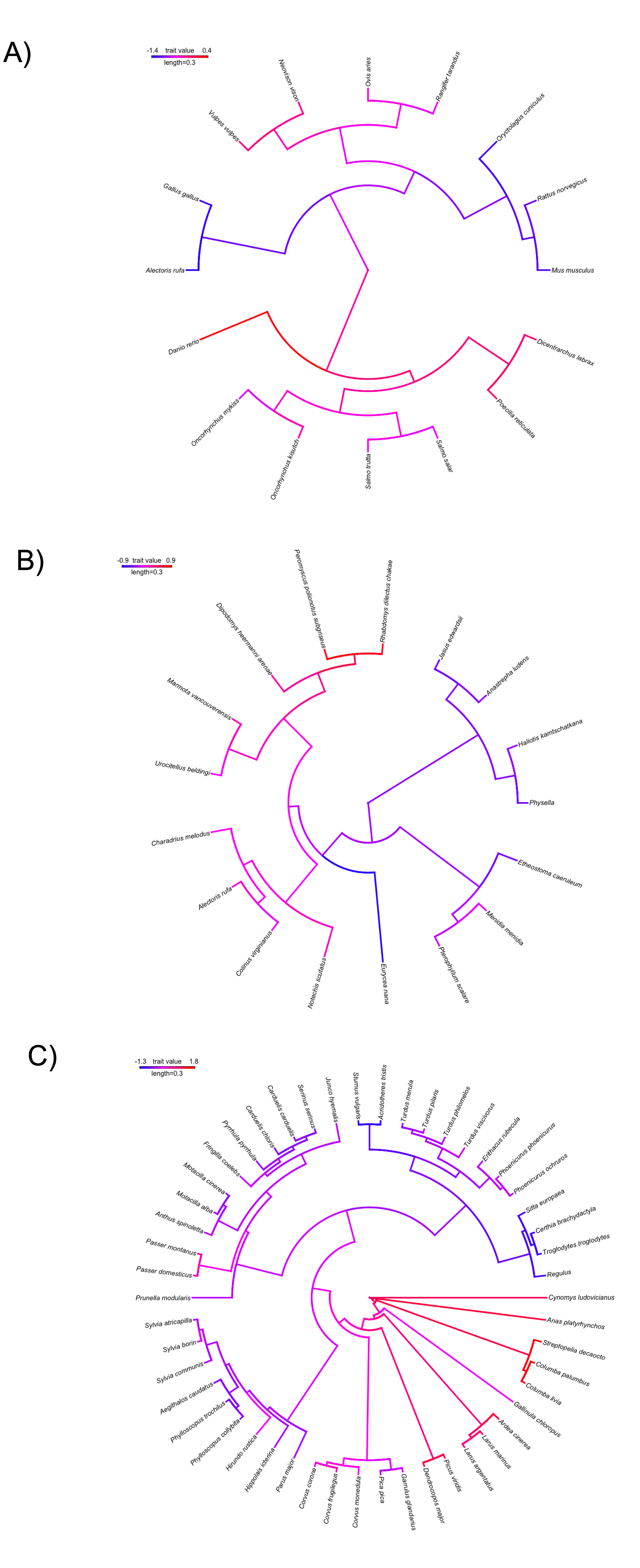

Supplement: S4 Fig — Phylogenetic trees of animals used in the phylogenetic meta-analysis for (A) domesticated, (B) captive, and (C) urbanized species. All data and R code supporting the figure are available in S1C Data. (TIFF) [file pbio.3000818.s005.tiff]

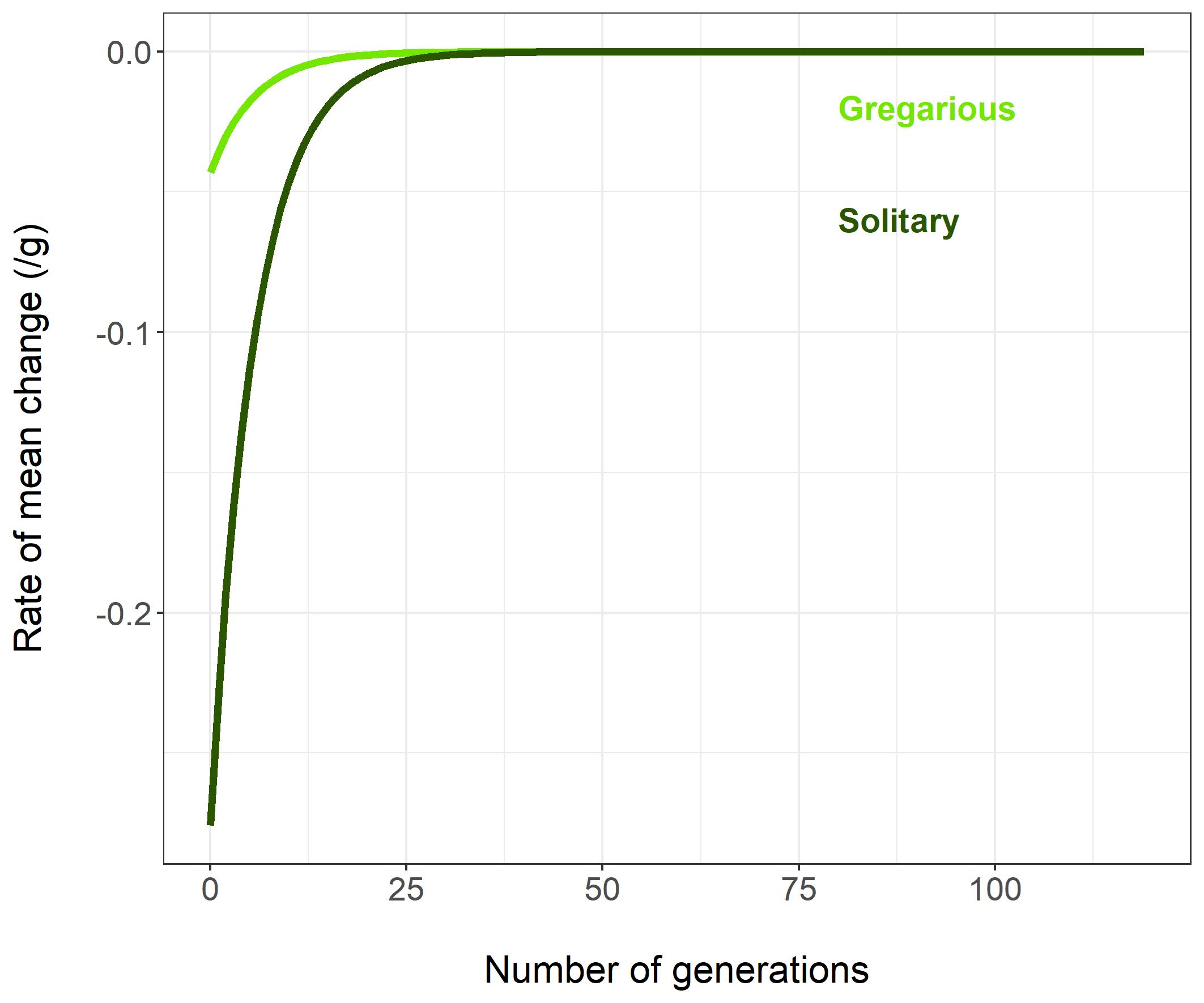

Supplement: S1 Data — All data for the meta-analysis, (A) initial data management, (B) data management, and (C) R code and data. (ZIP) [file pbio.3000818.s012.zip › S1 Data/C/Figure domes sociality deriv.jpg]

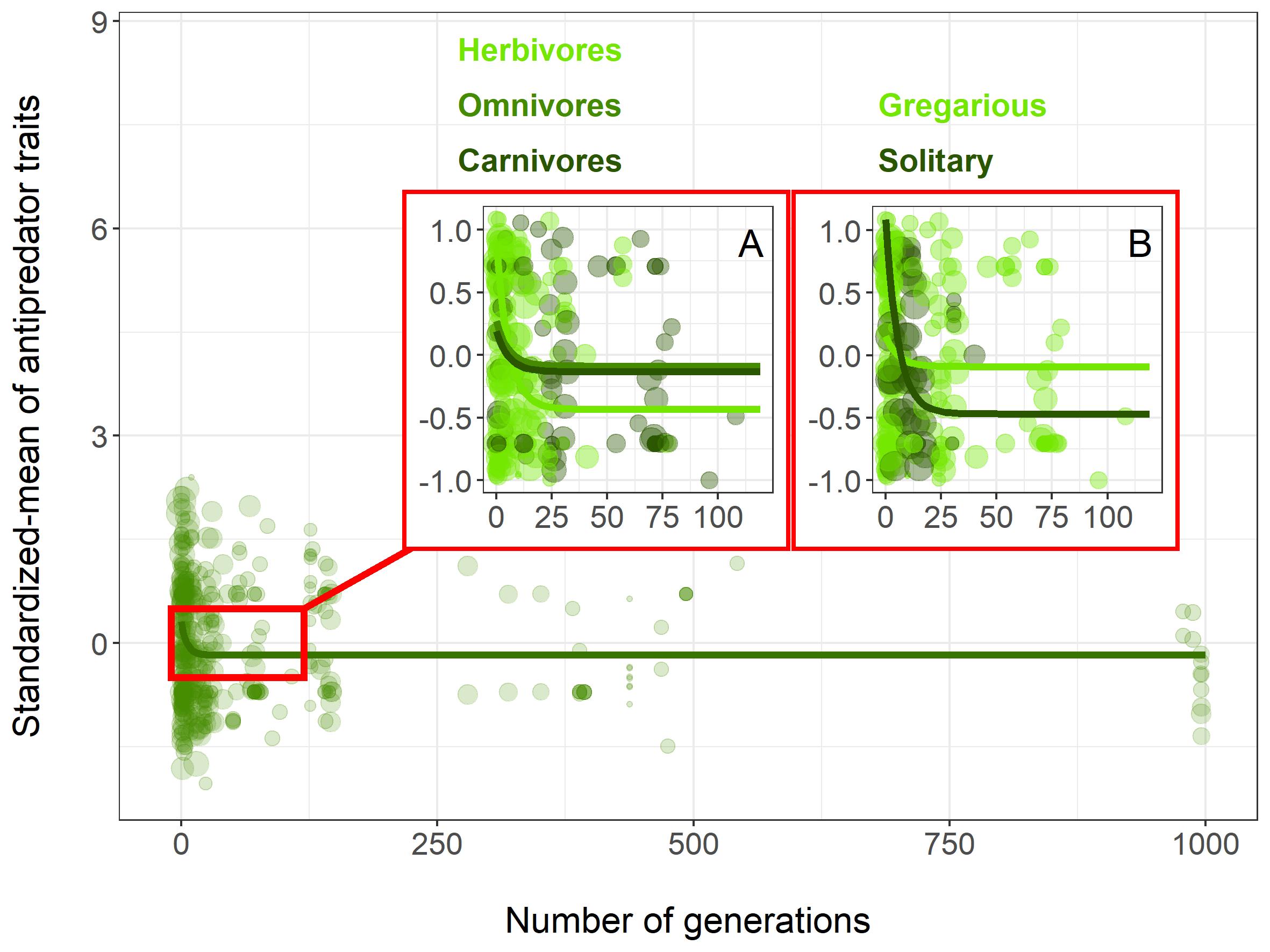

Supplement: S1 Data — All data for the meta-analysis, (A) initial data management, (B) data management, and (C) R code and data. (ZIP) [file pbio.3000818.s012.zip › S1 Data/C/Figure_LHT_domes.jpg]

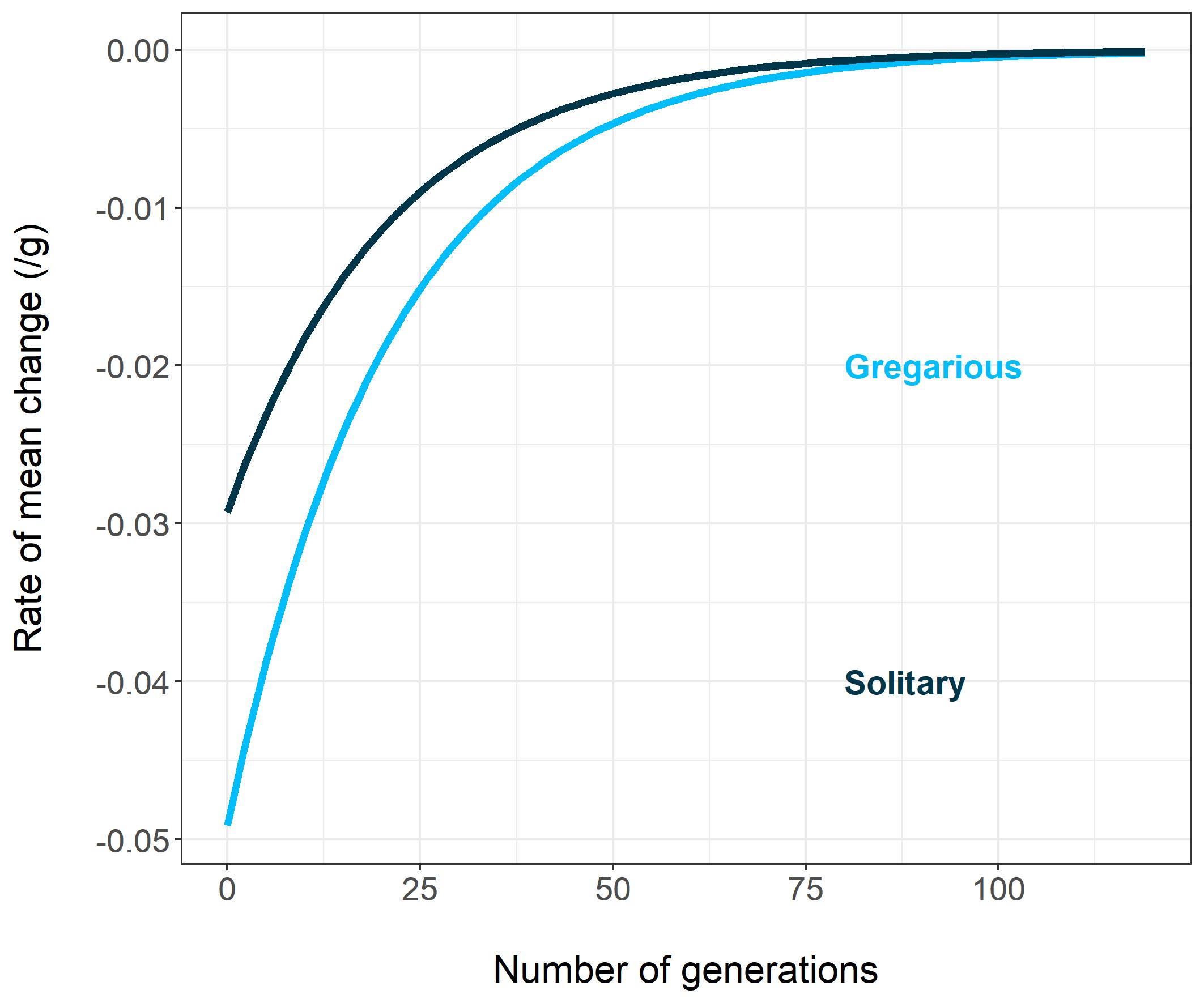

Supplement: S1 Data — All data for the meta-analysis, (A) initial data management, (B) data management, and (C) R code and data. (ZIP) [file pbio.3000818.s012.zip › S1 Data/C/Figure urban sociality deriv.jpg]

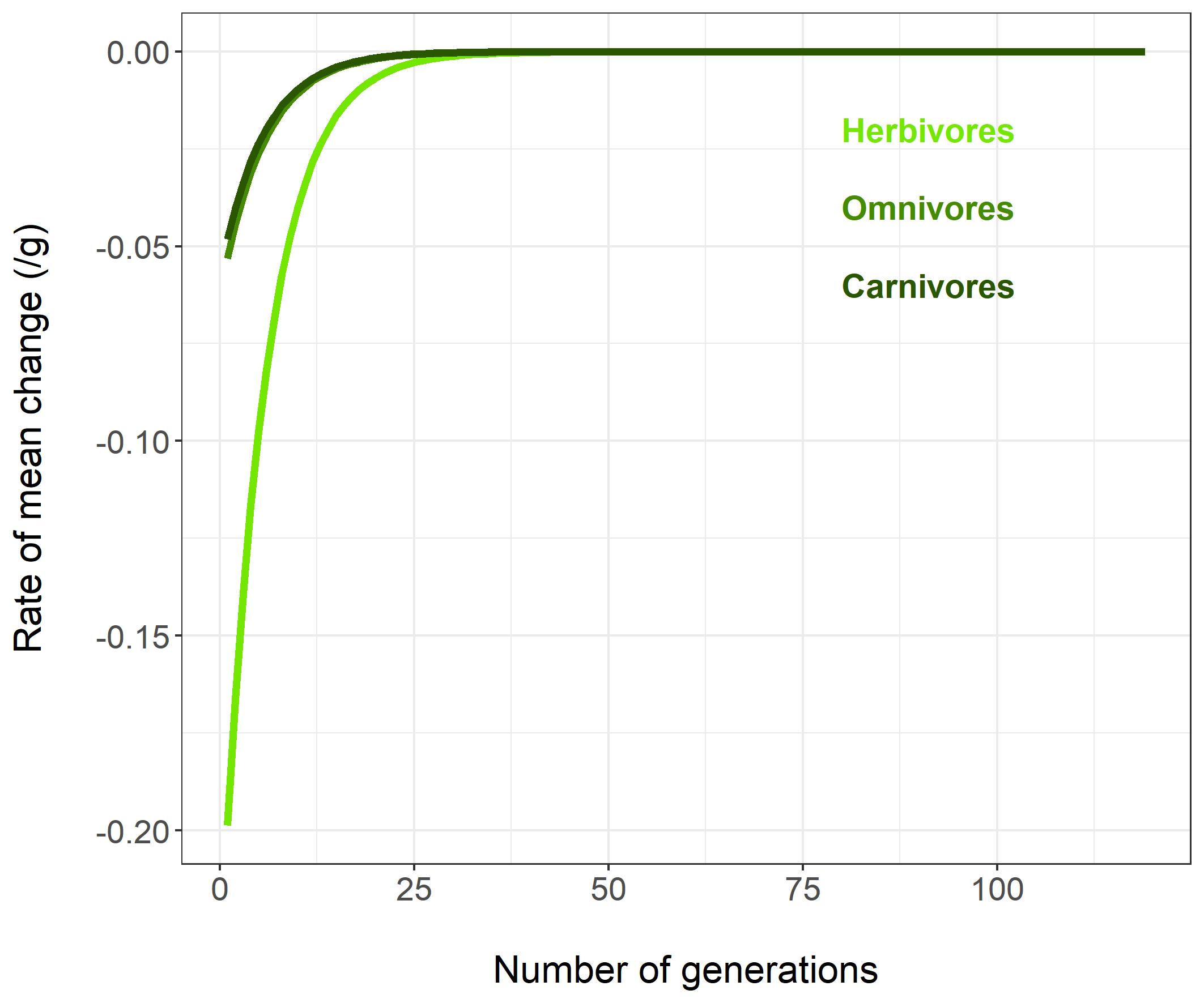

Supplement: S1 Data — All data for the meta-analysis, (A) initial data management, (B) data management, and (C) R code and data. (ZIP) [file pbio.3000818.s012.zip › S1 Data/C/Figure domes foraging deriv.jpg]

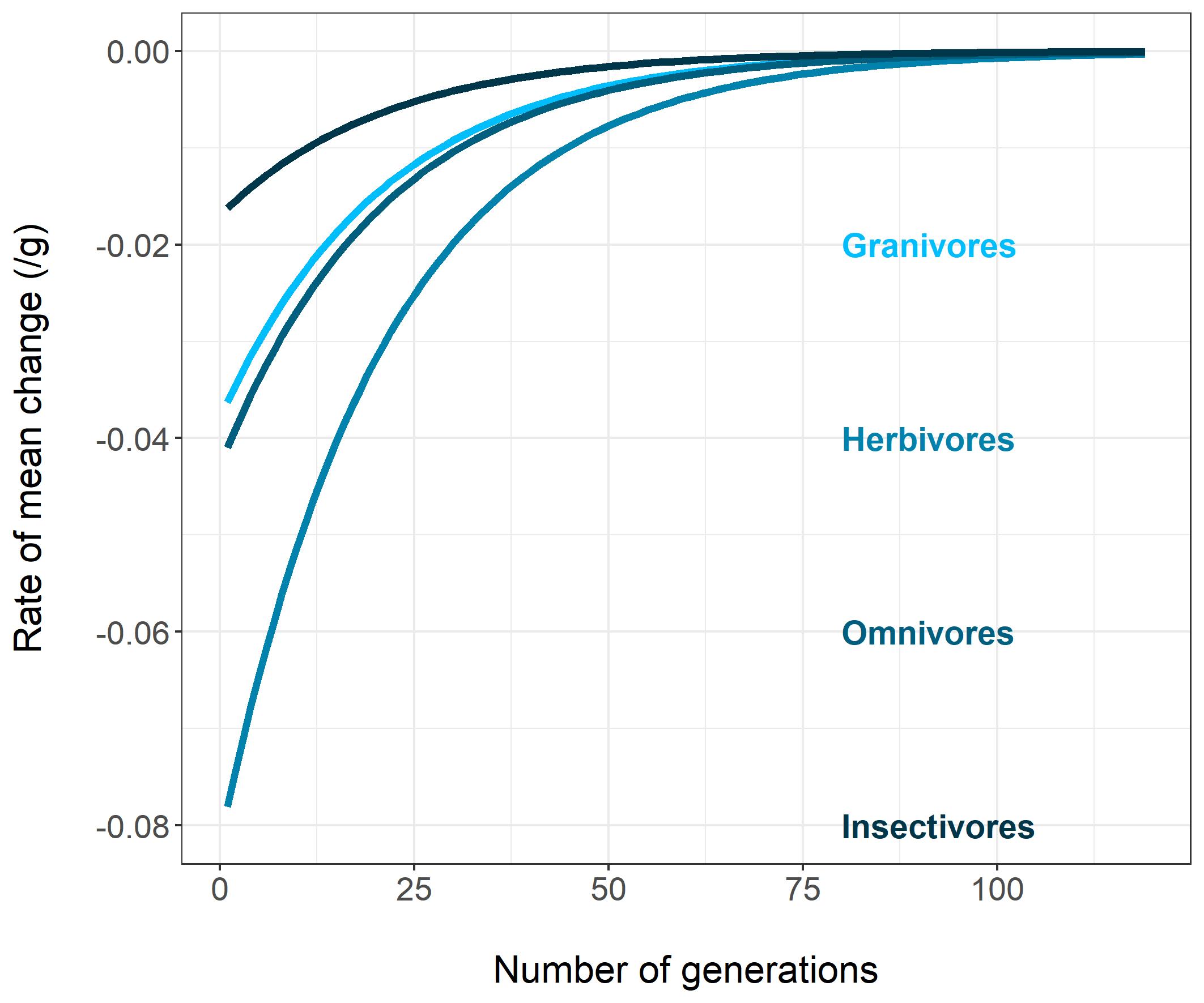

Supplement: S1 Data — All data for the meta-analysis, (A) initial data management, (B) data management, and (C) R code and data. (ZIP) [file pbio.3000818.s012.zip › S1 Data/C/Figure urban foraging deriv.jpg]

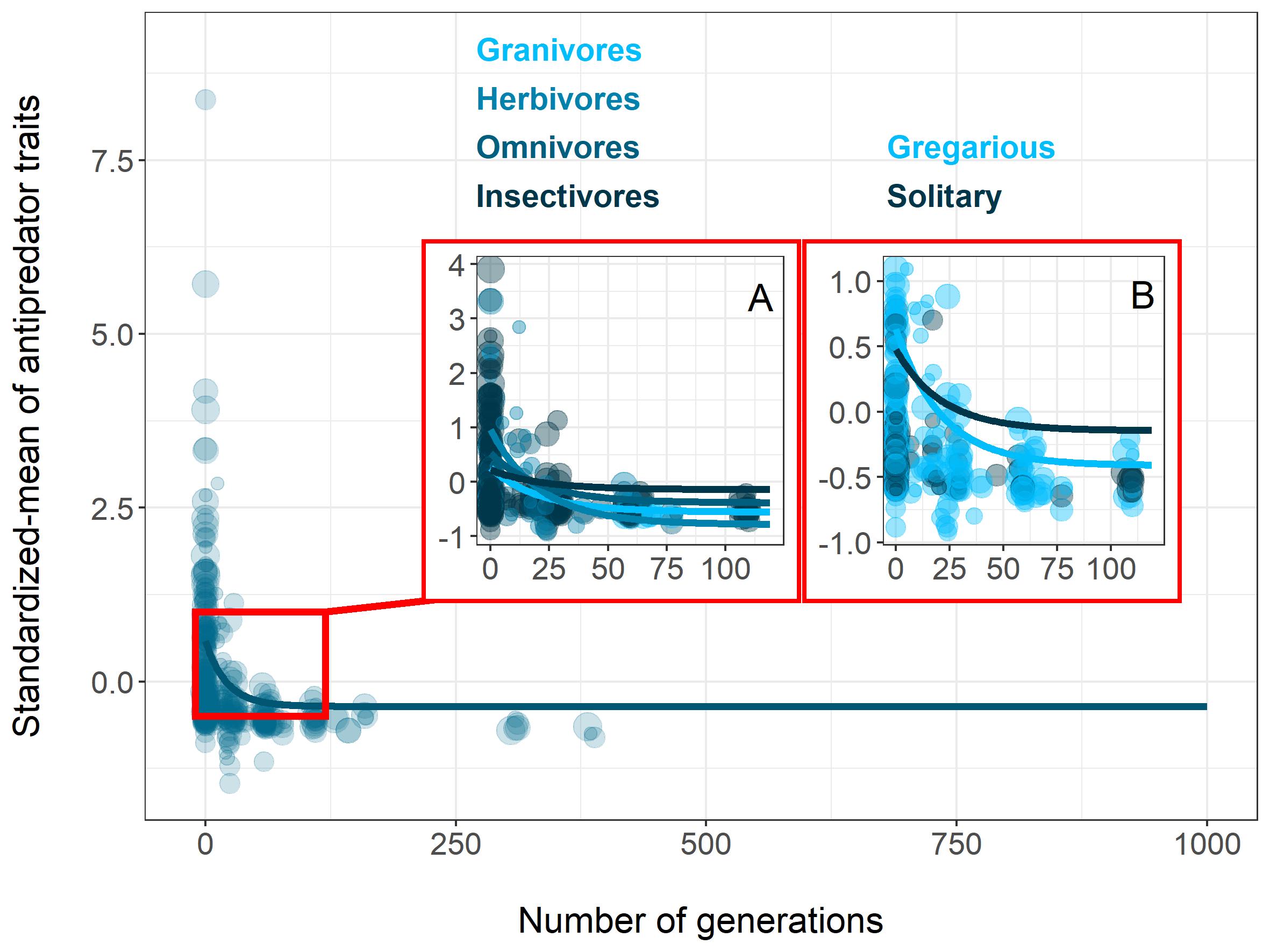

Supplement: S1 Data — All data for the meta-analysis, (A) initial data management, (B) data management, and (C) R code and data. (ZIP) [file pbio.3000818.s012.zip › S1 Data/C/Figure_LHT_urban.jpg]

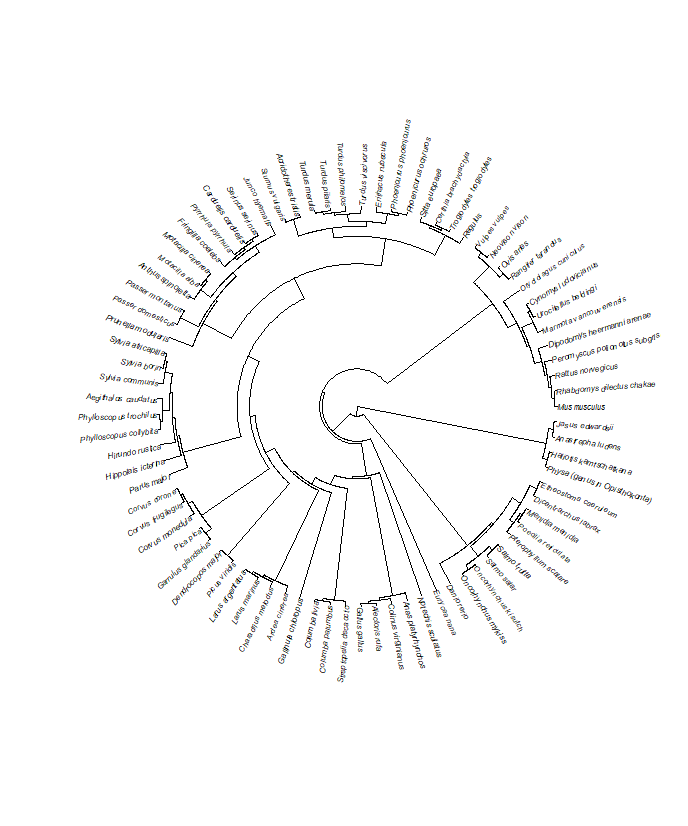

Supplement: S1 Data — All data for the meta-analysis, (A) initial data management, (B) data management, and (C) R code and data. (ZIP) [file pbio.3000818.s012.zip › S1 Data/B/Phylogeny All.tif]
