# Supplementary material for: Evolutionary dynamics in the Anthropocene: Life history and intensity of human contact shape antipredator responses
Source: PLoS Biol. 2020 Sep 22;18(9):e3000818. doi: 10.1371/journal.pbio.3000818 (PMC7508406; doi:10.1371/journal.pbio.3000818)
Supplement: Attachment — Submitted filename: Response to reviewers.docx [file pbio.3000818.s013.docx]

Dear Editor,

Thank you very much for giving us an opportunity to revise our MS, “Unravelling the evolutionary dynamics of antipredator traits following exposure to humans”. We have taken the very constructive suggestions from the reviewers to heart and have made substantial changes to address their concerns. This includes new analyses. We document the changes below and include two versions one with track-changes and one without to facilitate the review. We hope that with these revisions you will find the paper acceptable for publication. We are certainly willing to make additional edits under your guidance to further improve the paper.

On behalf of my co-authors,

Benjamin Geffroy

Reviewer #1:

This MS addresses an important topic in an era of human transformation of the planet.  Many species have traits that can be subject to new evolutionary ecological pressure.  Developing an understanding of the consequences of those new evolutionary ecological pressures on species is necessary to understand the fate of biodiversity.  

The MS reports on a meta-analysis of the loss of anti-predator responses of prey species subject to different kinds of human agency and pressure (i.e., urbanization, captivity, and domestication).  It compares rates of loss of anti-predator responses across numerous behavioral and physiological traits among the three different human agencies/pressures. It concludes that there is rapid loss in trait responses associated with all three human agencies. While the findings are potentially interesting, I find that I cannot judge whether or not the results are scientifically robust.  This is because the presentation of methods and data are inadequate to understand the reliability of the data used in the analyses. At the very least, the MS fails to meet my litmus test that the procedures used in the MS are sufficiently explained that I could repeat the study independently without consulting the authors.

**Response:** We thank the reviewer for these comments. We have revised the material and methods section to improve the clarity and to better explain exactly what we have done. Most importantly, we now provide the raw values we obtained after the initial literature analysis and also share all of the R code used to get to our final results (data management, statistical analysis and figures). In the process of doing this we caught a mistake. We detected some “0” in the coefficient of variation (CV) column that should have been recorded as NA for some captive animals. This does not change the conclusions, but slightly changes the pattern reported for captive animals. We have updated our results to report the new conclusions.

The MS states (beginning line 235) that there were 61, 59, and 107 studies that provide trait response data for urbanization, captivity and domestication respectively.  But later (beginning line 243) the narrative states that 417, 206 and 437 means were estimated from those studies.  Clearly there is a lack of independence in the data here.  But it is unclear what that lack of independence stems from.  Is it due to time series of trait changes presented in the different studies; or is it due to multiple traits measured in the same study; or is it a combination of the two?  This needs clarification.

**Response:** It is indeed a combination of the two, some studies provided multiple traits, and all have at least 2 generations per trait. As mentioned in the methods section, we selected only studies with at least 2 generations per trait. We now clarified this in the methods section by providing the mean (±SD) number of generations per trait (4.7±4.5) and the mean number of traits per study (1.6±1.1) line 302-303.

As well, how the lack of independence is dealt with in the data analyses needs better explanation.

**Response:** The lack of independence related to multiple values within studies is dealt with by including a random term in the mixed model. This statistically accounts for any repeated measures. We added this explanation to the text (Lines 371-373)

Moreover, it is unclear to me how you can estimate 437 means for domestication yet have 527 standard deviations.

**Response:** This is because 90 values (=527-437) come from studies that investigated the effect of a selection process designed to increase anti-predator related traits for research purposes. Those studies which actively selected for a trait in contradiction with the process of domestication (more for experimental purposes) were therefore excluded from the analysis of the domestication effect on antipredator traits. Nevertheless, the variability between individuals was still worth analyzing and therefore retained in the main dataset. We explain this in the text (lines 297-298).

It would also help to understand how the studies from which the meta-data were extracted were generally done. That is, were they experiments that challenged prey with risk or risk cues relative to proper controls? Were they field or laboratory-based studies?  How did the experiment risk or risk cue compare in magnitude to risk under "wild" conditions.

**Response:** We retained all values of antipredator traits whether they were obtained in a risky situation or not, but each was considered as an individual trait. A large majority of the studies related to domestication were performed in a very controlled environment (laboratory conditions or rearing environments). For urbanization, most studies provided information from an open environment. The distinction between field or laboratory-based studies is applicable for captivity and we now include a new column in the initial dataset providing this information.

How was the number of generations of human exposure determined for a species used in a particular study?

**Response:** It depended on the context. For captivity most studies provided the number of generations (or at least a range, and we then took the average), and when this value wasn't provided, we contacted the authors in order to obtain it. For domestication most of the time it was provided in the paper. When the value was not available, there was at least the time spent with humans in the publication, so that we were able to calculate an estimated generation time based on the average age of maturity of the focal species. We did this calculation for 28% of the initial values for domestication. Concerning urbanization, we used the time since colonization divided by the average time to maturity.

Analyses of trait response effects use different kinds of traits for urbanization, captivity and domestication (Table S1).  Analyses for urbanization seem to be based exclusively on behavioral traits.  The other two are based on behavioral and physiological responses combined.  How do we know that conclusions about differences in the magnitude of trait responses is due to the form of human agency (as concluded in the MS); as opposed to the use of different kinds of traits, or even an imbalance in the relative number of physiological and behavioral traits used for each form of agency that may confound the comparison? This concern stems from the observation (line 306) that behavioral and physiological trait responses can each have significant effects on mean and variance.

**Response:** Yes, in fact the type of trait (physiological or behavioral) has been included as a random factor “trait type” in the meta-analysis to address this issue (line 372). The variance linked to the type of behavioral trait is indeed very high in the all context analyses, but even after accounting for this effect, we find similar trends, which is really interesting.

Beyond identifying broad taxonomic groups, the narrative in the MS is vague on what particular species were used in the data analyses and where the data for each species falls along the generation time continuum (x-axis).  My concern is that with any species that has at least a generation time of a year or more, how does one get data for 120 generations (let alone 1000 generations) when it is unlikely that the scientific studies presenting the trait data likely weren't even published that long ago.  Perhaps the data presented in Fig. 2 are cross-sectional, using different species and the number of generations that they have been associated with humans. But how do we robustly know that a species that has, say been associated with humans for 1000 generations, behaved differently before human contact?

**Response:** Comparisons between generations do not necessarily mean that we are making comparisons at different times. Two subgroups of individuals, one domesticated and one coming from the wild can be tested at the same time. Thus, we don’t fully understand the reviewer’s concern here. For some species, the difficulty can indeed reside in identifying generation 0. This is the case for dogs, which is one reason why we removed dogs from the analysis. But for most other species, this was not an obvious problem. Even zebrafish, which have been domesticated for many generations, can still be found in the wild and therefore it is sensible to compare wild-type versus domesticated stocks.

Also, if data for different species fall on different points along the generation time axis, how do we know that data for the heightened trait responses when species have only recently been associated with humans (data near origin in Fig. 2) aren't for species that are naturally more apprehensive to predators than other species that may fall in the tails of the axis?  What I am driving at is how are the data for each of the 16 domesticated, 16 captive and 50 urbanized species distributed along the x-axes of Fig. 2?   All of this is to say that I have concerns about how the study "controls" for human effects, in the sense of robustly knowing what the species' trait responses were before being exposed or subjected to human effects and what the responses were gradually over the many generations associated with humans.

**Response:** The general trend that we found is statistically significant only because multiple species were studied. Focusing on a subset of species would not provide enough information on the global trend for the reasons you mentioned. Please note that we included species as a random factor in the model, to specifically account for differences between species. If this variability was too high, we would not have a significant effect. Thus, we do not believe that this is an insurmountable issue and we believe that we have adequately addressed it.

The sources of the data are presented in the supplemental file ALL.csv.  But the information in that table is not presented transparently nor is there an adequate description of what all of the column headers mean. Hence it is very difficult to verify the sources of the data.

**Response:** We now provide all initial data and R code in the online version. Anybody can now redo the analysis starting from the raw files.

Additional editorial comments

Line 67—problem with logic.  The sentence states that prior to human contact species have been under strong selection to avoid predation.  This sentence could be interpreted this way: If humans didn't contact the species, how do we know that they were or weren't under strong selection, because there were no humans around to measure things.  Please consider rephrasing.

**Response:** The abstract has been comprehensively re-written.

Line 71—expand on the conceptual ideas from evolutionary biology for why knowing patterns of variation in anti-predator responses is even important.

**Response:** Addressed.

Line 74-75—this explanation of "human shield" is inaccurate.  The original conception says nothing about reduction in the number and diversity of predators.  The original conception merely states that prey that associate with humans or human infrastructure may be protected from predation because of the deterrent effects of human presence. In the human shields idea, predators merely spatially disassociate from prey that are near humans or human infrastructure.

**Response:** Clarified; thank you!

Line 75-76—this sweeping claim that predation risk is essentially eliminated for domesticated animals is also inaccurate.  Many domesticated cattle and sheep are subject to predation—the cause of much human-carnivore conflict (hence predation pressure is not relaxed)—and those domesticated animals also often exhibit anti-predator behavior. This statement needs some qualification.

**Response:** Valid point; modified.

Line 80-85—the narrative says that the study will test the "relaxed-selection hypothesis".  But in the discussion, it also invokes phenotypic plasticity as a possible explanation for trait changes.  This means that the study is not truly testing for relaxed selection—in fact as described, the data cannot speak to this because it would require conducting reciprocal transplant experiments to test for adaptation.  The study can only say that the trait responses to predation challenges seem to diminish over time either from phenotypic plasticity or form altered selection.

**Response:** We have modified our prose to recognize the several processes that could create this change.


Reviewer #2:

The authors, an all-star group of behavioral ecologists with a history of studying antipredator behavior and/or applying behavioral insights to conservation biology, conducted a meta-analysis looking at changes in the mean and coefficient of variation in antipredator traits (I will call it fear) following exposure to humans in the context of domestication, captivity and urbanization.  This is a valuable endeavor.  Taking a devil's advocate view, however, one could say that showing that animals have generally become less fearful after generations of exposure to humans is obvious, and even the fact that this has happened particularly rapidly in domesticated animals seems obvious.  Along these lines, the authors' suggestion that "conservation scientists should pay attention to effects of a predator-free captive environment on antipredator traits, especially if captivity aims at a future reintroduction" is something that is already an established part of conventional wisdom, and
the notion that they might need to be taught or exposed to predators is also well known and widely discussed.  Still, although some of their main results or conclusions are obvious, it is reasonably valuable to test them with a meta-analysis.

**Response**: We agree that the decrease in mean anti-predator traits over generations was indeed expected, despite the very large number of species incorporated here and the variability in the type of human contacts. We nevertheless think that our main result is of great importance as it shows that interacting with humans affects most species in a very similar ways in all three contexts (i.e. domestication and urbanization lead to the same evolutionary changes, just on a different time-scale). In addition, it was yet unknown 1) to what extent the intensity of human contact impacts those traits in the different contexts, and 2) how the variance evolves over generations. Our results are thus novel. Finally, and in response to the suggestions below, we further expanded our analyses and now have further insights into the drivers of these responses.

While it is useful to do a meta-analysis to confirm these obvious trends, to me, a more interesting analysis (i.e., one that could tell us things we didn't already know), might be one that explains the variation in responses over time: why did some captive or domesticated species show almost immediate decreases in fear, while others initially retained their fear and took dozens or even 100 generations to become more fearful.  The very rapid decrease in fear - where numerous examples exhibited the maximum observed decrease in antipredator traits (-1) essentially immediately (figure 2A) - presumably primarily represents plasticity.  Conversely, even among domesticated species, figure 2 also shows some that are still quite fearful after 60-100 generations!  Besides the three main types of exposure to humans, what explains this striking variation in initial or long-term response?  An obvious idea is that the initial rapid response reflects plasticity, while species that took dozens or more generations to become less fearful have relied more on evolution.

Does their meta-analysis yield insights on why some species showed rapid plasticity (immediate learning, developmental plasticity or transgenerational plasticity perhaps involving social learning) while others do not, but instead required many generations to evolve a loss of fear even when held in conditions with no risk?  Why did some taxa not evolve a loss of fear even after so many generations without being attacked?  Is this related to something about the organisms: their trophic level (do predators lose fear more rapidly than prey?), taxonomic 'type' (do 'higher' vertebrates learn (individually or socially) to be less fearful more rapidly than herps or invertebrates?), activity type (do more active species learn more rapidly than sit-wait species?), social system (do more social species learn via social facilitation more rapidly than solitary species?).  Or does it relate to ecosystem type (e.g., the openness of the habitat - others have noted the difference between dynamics in open water vs vegetated zones, or between terrestrial habitats with less vs more vegetation structure)?  Or, is it related to the type of captivity or domestication (breeding for productivity as opposed to for work)?  Perhaps the authors do not have enough studies to address these interesting questions, but they are the ones that to me, would make for a more exciting paper!  To be a bit more specific, I am picturing analyses of the residuals, the deviations from the fitted functions of fear over time.  I realize that these analyses may be non-trivial, but still, if the authors do not add these analyses to this paper, I could imagine asking for access to their data set to do the analyses myself.  I am very curious to know what factors might explain which species or situations exhibit more rapid vs slower responses to living with humans!  

**Response:** Thank you for this comment! This is indeed a very good point that by addressing improved our paper. We followed your advice and searched for the key life history traits you suggested. We completed a new table with the following life history traits (LHT): Longevity Max, Foraging Guild, Taxonomic Type and Social level for all species we had (we added this file along all the data used for our meta-analysis in the Supplementary Material). We searched for “ecosystem type”, but the variability was so high among species that we finally decided to discard it. We also provide in the new Life history dataset (Table S5) the reason why the species was domesticated (fur, food, etc.)(line 321-325). We then included all these fixed effects for all models (CV and means) and for each context (domestication, captivity and urbanization). We then fitted those models with only one LHT, and many LHT for the same variable (CV or mean) investigated. We then compared full models (4 LHT) to other models with different possibilities (3, 2 and 1 LHT) with always the maximum number of random factors (i.e., 4), and classified them based on their DIC using the MuMIn package (Line 378-386). Overall, we found that none of the LHT explained the evolution of CV over time. However, we obtained very interesting results for the mean. The decrease in the mean was largely explained by Foraging Guild and Social Level (both were significant) in domesticated species (line 128-142)(new Figure 3). The taxonomic type (alone) also appeared to impact how the mean decreased over generations of exposure to humans, but it was not conserved in the most parsimonious model where only Foraging Guild and Social Level were retained. The decrease of the mean observed for captive species was not significant and none of the LHT were significant either. Similarly to domesticated species, we found out that some LHT significantly explained the inverse decrease of the mean over generations of exposure to humans in urban areas. All LHT significantly (or were close to being significant) explained the decrease in antipredatory traits, when tested alone (line 128-142). Here again, we compared full models (4 LHT) to other models with different possibilities (3, 2 and 1 LHT) with always the maximum number of random factors (i.e., 4), and classified them based on their DIC using the MuMIn package. This resulted in a same pattern to that of domesticated species with both Foraging Guild and Social Level being significant in the same (best according to the DIC) model (new Figure 4). This has now been included in 2 new figures and model comparisons have been added in a new Table. Again, we thank the reviewer for this highly generative comment.

In this context, it is worth noting that a classic paper by Hendry et al. 2008 Molecular Ecology 17:20-29 examined effects of humans on phenotypic change and found that many examples exhibited very rapid change that suggests that plasticity is very important.  Perhaps I did not look carefully enough, but the authors did not apparently cite this important, relevant paper.

**Response:** We agree that this paper is extremely relevant to our study and we have added a direct reference to it in our discussion (lines 168-170)

The fact that domestication produced a decrease in fear that reached an asymptote after only about 25 generations, but that the coefficient of variation continued to increase very substantially for another 30 generations or so seems difficult to explain.  Logically, I can understand an initial increase in CV even before the mean has decreased much; that is, I can imagine a population of fearful animals adding (under relaxed selection) more and more less fearful individuals with initially little decrease in the number of fearful ones, but why would the CV continue to increase even after the mean has become substantially less fearful?  The authors discussed this, but I did not find their discussion illuminating.

**Response:** This is indeed an intriguing result, and we spend some time to try to understand it. We can’t be completely sure of the biological reasons behind this phenomenon, but we have one main supposition that would be worth testing. The mean changes overtime because of 2 processes probably staggered, 1. the reduced selective pressure on shy individuals (more fearless individuals), 2. The new selective pressure or the adaptation to the new environment with individuals potentially counterbalancing process 1.

The mean inverse decrease would then maybe hide 2 underlying curves, 1 inverse and 1 logistic that we can’t really separate because of a lack of data but creating the weibull when looking at the CV.

Figure 1 is not easy to interpret.  The y-axis is the occurrence of the two extremes with the black line representing fearful individuals and white lines the fearless ones.  Since the black line starts at only an intermediate level (as opposed to a maximum level), I am guessing that by "occurrence", the authors mean the absolute abundance of the types and not the proportional (relative) frequency.  Please clarify the meaning of "occurrence".  To me, it seems unfortunate to simplify the world into two types.  The animal personality literature has been criticized for implying that there are two behavioral types (here, high fear and low fear) as opposed to the reality of a continuous distribution.  Perpetuating this problem seems like a bad idea.  With urbanization and captivity, the authors suggest that the fearful animals decrease in abundance, while after a while, fearless ones spontaneously appear and increase in prevalence.  But in domestication, the fearful animals do not decrease, but instead also increase rapidly in occurrence.  Do the authors really mean to suggest these patterns of change for these two behavioral types?  If nothing else, they should explain their logic behind the patterns in figure 1 either in the legend or the text.  In all three situations, the fearful ones blend into fearless ones (the color of the line goes from black to grey and the line then joins the white line; perhaps this reflects plasticity?), as opposed to the frequency of fearful ones (the black line) continuing to decrease over time.  Is this really what the authors have in mind for the dynamics of trait change?  Some explanation would be useful.  With urbanization and captivity, overall population size continues to increase more or less exponentially (though faster in captivity) while in domestication, it asymptotes.  Do the authors really mean to suggest these contrasting population dynamics?  The human shield effect is shown to gradually increase smoothly with urbanization and captivity, though more quickly in captivity, as opposed to an abrupt increase in domestication.  In all three cases, the color intensity suggests that after some time, the human shield gets equally strong.  In contrast, based on the text, one might think that all three situations change abruptly (not gradually over time) from 'natural' to human-altered, but that the magnitude of the human shield differs among situations.  

The bottom part of figure 1 is labelled as "cumulated variance".  I am not sure what this means.  Is variance piling up over time - are the authors suggesting that it is meaningful to add up variance across time?  What does that mean?  Or, perhaps they just mean expected variance - and the general notion (as stated on lines 84-85) that with relaxed selection, the population might harbor greater variance in fearfulness.  However, that would seem to suggest greater variance allowed with a stronger human shield, but the graph shows the prediction that with a stronger human shield, the cumulated variance increases faster, but to a lower level.  This prediction needs further explanation either in the text or in the figure legend.

Overall, I suggest a complete reworking of figure 1.

**Response:** We have deleted figure 1. It seems to be confusing and we decided it is not required. Rather, we now better highlight our hypotheses in the introduction. We hope that our figures (both in the text and the supplemental ones) are now legible and help show the pattern of responses.

A few minor points

Please standardize spelling: urbanization vs urbanization.
**Response:** Done.

Line 70: odd to refer to antipredator behavior as being at a "context-dependent equilibrium".  While the behaviors are no doubt context-dependent, they are not usually referred to as being at an equilibrium.

**Response:** You are correct; we deleted the reference to being in equilibrium.

Line 241. The authors removed 90 examples of where animals were selected for increased fear or aggressiveness.  It is probably OK to remove these examples from THIS paper, but it would be interesting to contrast the efficacy of responses to selection for reduced vs increased fear.

**Response:** All raw data from the initial extraction will be included in the final submission and will be available online, so that anybody can use them for other purposes.

Line 256.  Data were standardized.  In that context, why do domesticated animals appear to have a much higher mean level of fear in generation zero than captive or urbanized species?  Statistically, the fact that domesticated animals were more fearful when first brought into captivity seems to explain why they exhibited a larger, more rapid decrease in fear.  But shouldn't all groups have started out with a mean fear level close to zero?  I am clearly not understanding something about what figure 2 shows.

**Response:** Data were indeed standardized by dividing by the standard deviation within traits and studies. This allows to work with traits on a same scale and unit. This procedure does permit the relative comparison among traits, studies and context but does not affect the speed of decrease or the shape of the curve. The comparisons have therefore to be made on the derivatives and overall observed curves (and associated significance). Consequently, we cannot say that the mean starts higher for a context compared to another or that it ends lower than another but rather that it decreases more quickly.

Overall, this is an interesting and valuable paper, but could be made somewhat more clear (in particular, figure 1), and much more valuable with additional analyses.

**Response:** Thank you for your generative comments! We believe that by addressing them we have greatly improved the MS.


Reviewer #3:

I think that the strengths of this paper relate to the phylogenetic meta-analysis. That strikes me as a very novel contribution to the broad field of study which is seeking to understand how animal communities are changing in light of human behavior. However, the scope of the study is not well-presented leaving the reader to wonder what is the point.

The paper, at present is a bit under-referenced. There is so much literature on this broader topic, I was surprised to see that the authors only cited 44 papers and just 14 in the introduction. However, this may be because of formatting requirements in PLOS Biology. If so, then please disregard my comment. However, if there is not a strict reference limit for this paper, then the introduction, in particular, feels very under-developed. Several paragraphs in that section, for instance, are just 3-4 sentences in length. It seems that the authors are taking for granted that the readers automatically 'get' what they are proposing to study. I get it, but would like to be supported in the narrative so that my attention continues to be allocated to that which the authors want me to understand.

**Response:**. Thank you for this comment. We have expanded our introduction and discussion and have added 26 more references to the manuscript.

More broadly, it is unclear what new information derives from this manuscript. The presentation of the role of this paper by the authors makes it challenging to interpret the values of the results. We already know that urbanization and domestication reduce the intensity of anti-predator traits. The authors admit this in the discussion section when referencing the broader literature. What would be the consequences of reduced anti-predator traits in captive animal populations? After all, animals in captivity will likely never be released into the wild. Thus, there is no real need for anti-predator traits and the reduction of those traits may actually present advantages to people that manage captive facilities (i.e. increased docile nature). Thus, the recommendations emanating from this paper seem odd. The authors write on line 196 that society might consider "intentionally exposing animals in captivity for conservation purposes to predators or predator-related cues." How would that it any way be a conservation-oriented decision? Furthermore, there would be profound ethical dimensions that would be necessary to unpack before a decision like that could be made.

**Response:** A major justification for zoos and aquaria is captive breeding animals for subsequent release into the wild. In addition to these breeding facilities, many conservation programs have bespoke captive breeding facilities to recover a species from the edge of extinction and later reintroduce animals into the wild. We cite key references that talk about the problems of this—namely the loss of antipredator behavior in captivity. (lines 269-282)

Line 62: Undoubtedly, all animals have been affected by human disturbance on planet earth. However, it would seem that the structure of this sentence is most applicable to large vertebrates. I recommend that the authors look specifically at the generality or specificity of their writing in recognition of the fact that sentences like this don't well apply to all animals.

**Response:** We have added caveats as required in many places in the MS (for example, lines 58 and 76).

Line 67: This selective pressure persists in the Anthropocene. I believe that authors are suggesting that the strength of these effects may modulate in line with sustained human interaction. But that principle is not well-conveyed in this sentence.

**Response:** Modified.

Line 70: Do the authors mean "allocation of antipredator"

**Response:** Changed to ‘of’.

Line 67: After reading this paragraph, I would recommend that the authors present this information in the absence of human interaction (i.e., the functioning of non-human predator-prey systems). Then the transition to the modulation of these interaction effects via humans can be presented in the following paragraph.

Line 74: Some setup identifying that the interest here is in three human things (as done so in the abstract) including urbanisation, captivity, and domestication is needed. Furthermore, these are immensely complex relationships that will be stronger for some animal species over others. The reader needs to understand which species the authors are planning to focus on. The authors also switch between urbanisation and urbanization. There is a need to standardize.

Line 84: This is the first suggestion of fear-related traits. The reader has been prepared to think that the focus is on anti-predator traits. Via that logic, the reader is thinking that the focus of this paper might be on prey species and perhaps large ones that are more likely to be affected by humans. However, fear-related traits could also apply to predator species. Thus, I am not quite confused. The authors need to help the reader understand what they are focusing on and describe how anti-predator and fear-based traits vary.

Line 95: The authors need to remind the reader which human influences (urbanisation, captivity, and domestication) they are referring to. Also, here again, we have indication of confusing variation in the terminology used by the authors as they once again refer to antipredator. Now we have antipredator traits, antipredator responses, antipredator phenotypes, fear-related traits, high fear, low fear, and fearlessness. The authors really need to standardize their presentation so that the reader can understand what they are trying to do.

**Response:** To address the four comments above, we have carefully gone through the introduction, making modifications to better clarify our message. We believe that it now clearly lays out the issues and questions that we address in the paper.

Figure 2: When the authors say 'interactions with humans' do they refer to urbanisation, captivity, and domestication? Interactions with humans is extremely broad and the magnitude of effects would be predicted to be highly variable.

**Response:** We refer to, and indeed plot, all three in our figures.

Line 129: Which 'contexts'? The authors need to do a much better job of developing a narrative flow to help the reader interpret this manuscript.

**Response:** We hope that with the substantial revisions our paper is easier to follow.

Line 146: I fail to see how domestication would result in a 'complete reduction in predation risk.' Many domestic animals still live in systems that are under the risk of depredation. Take for instance, the vast literature associated with human-carnivore conflict built upon carnivore depredation of livestock. Thus, the inference made by the authors on the next sentence is simply inaccurate.

**Response:** We emphasize that it is often reduced or may be eliminated, but can also still be present, and added a reference to support this (line 88-90). Palmeira FBL, Crawshaw PG, Haddad CM, Ferraz KMPMB, Verdade LM. Cattle depredation by puma (Puma concolor) and jaguar (Panthera onca) in central-western Brazil. Biological Conservation. 2008;141: 118–125. doi:10.1016/j.biocon.2007.09.015

Line 172: Aquaria? Do the results apply to aquatic species? How so? Again, it is very difficult to interpret which species this analysis is applicable to.

**Response:** We hope that these ideas are broadly generalizable to the situations where are assumptions are met. We have indeed several fish species in our analysis for captive and domesticated animals as shown in Figure 1 & 2. Bringing a species of fish into a predator free tank is similar to bringing hamsters into predator free laboratories or pet stores. We hope that this is sufficiently clear in the text.

Line 183: It is the authors responsibility to describe the implications of their results. They mention 'drastic consequences' that might emanate from the knowledge within this study. What are those?

**Response:** We expanded and modified our prose and eliminated ‘drastic’ (lines 236-240).

Line 196: Why would society be inclined to retain anti-predator behaviors in captive animals via exposing them to fear? What would be the ethical implications of such a design? I fail to see the logic here.

**Response:** There’s a lot of conservation literature that refers to this. We’ve cited several papers that are relevant to this topic here (lines 223-233; 243-254).
